# Fatal Mesenteric Ischemia Induced by Synthetic Cannabinoids: A Case Report and Literature Review

**DOI:** 10.1155/2017/6964078

**Published:** 2017-12-18

**Authors:** David Hakimian, Orr Tomer, Nurith Hiller, Samuel N. Heyman, Sarah Israel

**Affiliations:** ^1^Department of Internal Medicine, Hebrew University Hadassah Medical Center, Mt Scopus, Jerusalem, Israel; ^2^Department of Radiology, Hebrew University Hadassah Medical Center, Mt Scopus, Jerusalem, Israel

## Abstract

Worldwide use of synthetic cannabinoids (SCs) is rapidly increasing, in part due to the generation of numerous new compounds, sidestepping legal restrictions. Their detection using standard toxicology panels is difficult, due to their vast heterogeneity and lack of structural resemblance to cannabinoids. Sympathetic overactivity and arterial spasm play a role in some of the life-threatening reactions to SCs, such as coronary or cerebral vasoconstriction. Here we report a patient with repeated consumption of SCs that led to mesenteric ischemia and death. A 29-year-old man was frequently evaluated in the Emergency Medicine Department for recurrent transient crampy abdominal pain, associated with the use of the SCs colloquially known as “Mr. Nice Guy.” He was finally hospitalized with a protracted attack, associated with diarrhea and leukocytosis. Initial evaluation including computed tomography was unremarkable. Diarrhea and leukocytosis gradually resolved, but bouts of hypertension and abdominal pain occurred in association with repeated consumption of the SCs. On the fifth hospital day, the patient developed abrupt abdominal pain, associated with profound shock and signs of peritoneal irritation and succumbed within an hour. Postmortem CT scan was consistent with intestinal perforation most probably due to a nonobstructive mesenteric infarction. There was no evidence of a single vessel infarction.

## 1. Introduction

The clinical effects of delta-9-tetrahydrocannabinol (THC), the major active ingredient of cannabis, are mediated through action as a partial agonist of CB1 and CB2 receptors. Cannabinoid receptor CB1 is intensively expressed in the central nervous system, especially the hippocampus, but also in additional regions, such as the cortex, basal ganglia, and the amygdala.

Synthetic cannabinoids were first synthesized in the 1960s in laboratories exploring potential medical uses of compounds targeting cannabinoid receptors. They were not initially designed to be illicit drugs but, unfortunately, over the years, they were upgraded, synthesized, and distributed for illicit use [1].

SCs are a chemically heterogenic group of compounds that act as potent agonists to cannabinoid (CB) receptors (both CB1 and CB2). Most of the SCs (with an exception of HU-210) are significantly different in their chemical structures from cannabinoids. They may interact with other brain receptors, such as serotonin and N-methyl-D-aspartate (NDMA) receptors. SCs are often more potent agonists than cannabinoids, with reports of potencies ranging from 2 to 800 times greater than delta-9-tetrahydrocannabinol (THC). SCs are usually not detected by standard toxic screen panels but may be detected by gas chromatography and mass spectrometry [2]. Use of SCs results in markedly more toxic adverse effects than marijuana. The herbal product or plant used as the vessel for SCs delivery may also have hallucinogenic properties or may contain contaminants (such as caffeine, nicotine, and tramadol), often mixed with additional inert vegetable matter or unknown content to resemble potpourri or incense [3], which may all contribute to clinical effects and toxicity.

New SCs compounds evolve rapidly in the local and international illicit drug markets, in order to circumvent legal bans. Financial and technical limitations of forensic laboratories put law enforcement systems at a disadvantage in their continuous struggle against the illicit drug industry [4, 5]. In order to solve this problem, in May of 2016, the “Psychoactive Substances Act” was legislated in Israel. Following this act, for the first time, the illegality of a compound is to be based on its pharmacological action, rather than its chemical structure [6].

The clinical spectrum of SCs activity is rapidly expanding, as the chemical structures of SCs analogs are constantly changing, without regulatory measures for quality assurance, introducing additional toxic effects. The diverse mixtures and inconsistent doses provided by different suppliers can further cause heterogeneous, unpredictable, and challenging clinical presentations.

Mild intoxication often presents as agitation, delirium and weakness, frequently with gastrointestinal symptoms such as nausea or vomiting. Sympathomimetic features are common, including tachycardia and hypertension [7], but bradycardia and hypotension have been reported as well [8]. More severe symptoms reported over recent years include renal failure [9], acute ischemic stroke [8], seizures, arrhythmias, myocardial infarction [10], and even death [11–13].

Treatment is usually individualized and directed at the specific clinical presentation. Decisions regarding hospitalization, extent of observation, and treatment modalities are based on the symptoms, their severity, and comorbidities present [14].

SCs containing products are sold in Israel and abroad under various “street names” such as “Mister Nice Guy,” “Spice,” “K-9,” and “Lemon Grass.” These illicit drugs are available for purchase on the street, in kiosks, and online. They are usually consumed via smoking or as liquids vaporized and inhaled in e-cigarettes and other devices (liquid incense) or may be ingested [15].

Data from the largest therapeutic community for young male adults in Israel, provided by the European Monitoring Centre for Drugs and Drug Addiction [16], shows that, in 2013, 61% of patients were being treated for use of new psychoactive substances, principally SCs. Similarly, the number of SCs detected through the EU Early Warning System continues to grow, with a total of 160 synthetic cannabinoids having been notified to the EMCDDA as of December 2015 [17].

Here we report a case of a patient with recurrent episodes of mesenteric ischemia, induced by ongoing use of “Mr. Nice Guy,” ultimately leading to his demise.

## 2. Case Presentation

A 29-year-old man presented to the emergency department after one week of repeated bouts of crampy abdominal pain and diarrhea, which became bloody on the day of arrival. There was no fever, nausea, or vomiting.

He was evaluated several times in the Emergency Medicine Department during the previous three months, with various abdominal complaints that resolved after symptomatic relief without further investigation. The patient reported no additional symptoms or relevant personal or familial medical history. He worked as a bus driver, smoked cigarettes for many years, and did not drink alcohol regularly.

The patient reported daily usage of “Mr. Nice Guy” for the last few months but denied use of any other drugs or supplements. Consumption of SCs was via smoking, and he denied other means of administration such as oral or intravenous. This information was validated by collateral anamnesis of visiting friends.

In the emergency department, the patient was hemodynamically stable with initial BP recordings of 159/100 mmHg, regular heart rate of 99 bpm, and oxygen saturation of 99% on ambient air. Physical examination was unremarkable except for intense borborygmi and mild abnormal tenderness. Initial blood work showed leukocytosis of 20,000 cells/mcL with neutrophilia (80%) and normal hemoglobin (15.1 grams/dL). Biochemistry, including electrolytes, kidney function tests, liver, and pancreatic enzymes, were all within normal limits. Urinalysis was unremarkable, and urine toxicology screen was slightly positive for amphetamines and benzodiazepines but negative for other screened drugs including THC, opiates, and cocaine. Upon repeated questioning, patient once again denied use of any other stimulant including amphetamines, besides “Mr. Nice Guy.” White cell count increased to 28,000 cells/mcL six hours later, and an abdominal computed tomography (CT) performed without IV or PO contrast enhancement was unremarkable. Over the next four days the patient continued to complain of intermittent episodes of crampy abdominal pain with episodic diarrhea, followed by pain-free periods during which he strolled freely around the hospital, admitting that he was still smoking “Mr. Nice Guy,” provided to him by his friends. Additional toxic screens were not performed during hospitalization due to lack of the patient's cooperation. Leukocytosis gradually resolved. Blood, stool, and urinary cultures were negative and other laboratory data were unremarkable, with the exception of hypophosphatemia (0.5 mmol/L, normal = 0.8–1.4) and increasing C-reactive protein (22 mg/dL, normal < 0.5 mg/dL). During his hospitalization we abstained giving the patient medications from the family of benzodiazepines, potent opiates, and any medications which involve potential future addiction.

On the fifth hospitalization day, an abdominal X-ray, taken during a bout of abdominal pain, demonstrated dilated small bowel loops without features of intestinal obstruction or perforation, consistent with paralytic ileus. Later that day, the pain subsided and the patient requested to take a walk around the hospital, as our internal medicine ward regulations permit stable patients to leave their rooms. Obviously, hospitalized patients are not permitted to use any form of drug or medication not provided to them by hospital staff.

However upon returning from his walk, the patient admitted that he had once again consumed “Mr. Nice Guy” with one a friend who came to visit him. Routine checkup of vital signs revealed elevated blood pressure 180/110 mmHg with a heart rate of 120/min, without fever. Six hours later, he developed sudden extreme abdominal pain and was found to be in profound shock vomiting “coffee ground” material. Physical examination revealed abdominal guarding and rebound tenderness compatible with peritonitis, and laboratory evaluation disclosed hypoglycemia, lactic acidosis, thrombocytopenia, and worsening kidney function. An emergent surgical intervention was considered for an evolving abdominal catastrophe, but the patient quickly went into pulseless electrical activity with subsequent cardiac arrest.

**Figure 1:**
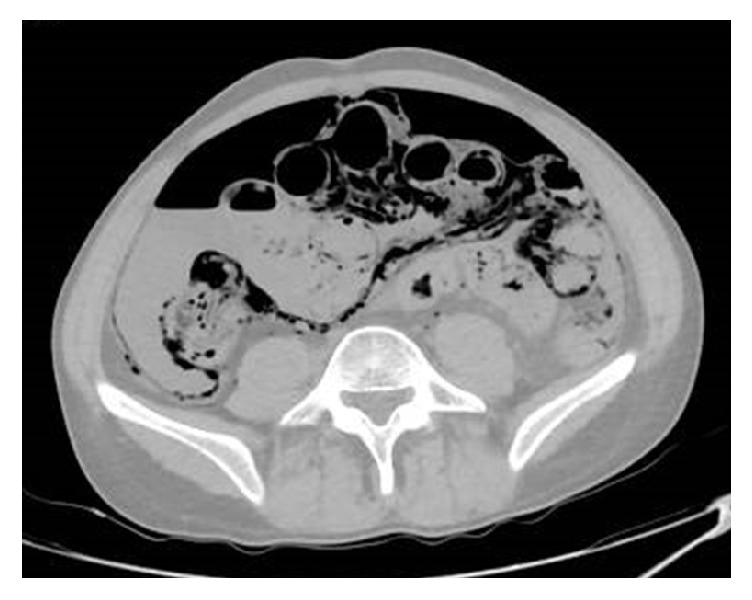
Postmortem CT, axial view, revealing pneumatosis intestinalis along the small bowel and right colon along with free air and unclear fluid in the peritoneal cavity.

**Figure 2:**
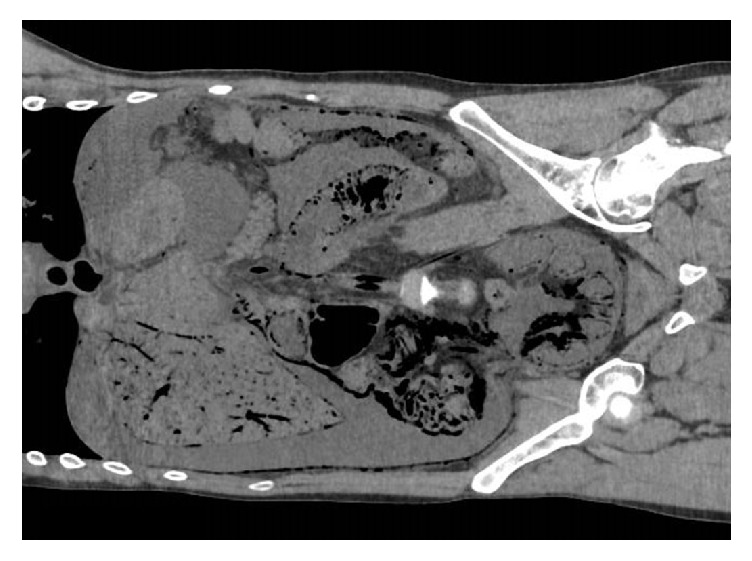
Postmortem CT, coronal view, pneumatosis intestinalis along the small bowel, right colon, and sigmoid, with gas in the portal system and blood vessels, with a large amount of unclear fluid around the liver and in the right paracolic gutter.

A request for autopsy was declined by the patient's family, but a postmortem CT scan one hour after death revealed extensive pneumatosis intestinalis of the entire small bowel, right colon and sigmoid, gas in the portal system, and blood vessels, with free air and unclear fluid in the peritoneal cavity (Figures 1 and 2). These findings were consistent with acute intestinal perforation most probably due to a nonobstructive mesenteric infarction (NOMI) as the cause of death. There was no evidence of single vessel infarction, rather of diffuse ischemia, possibly due to splanchnic vasospasm. Air in the aorta, right ventricle, and biliary tract were conceivably postmortem changes. Urinary toxic screen sampled during hemodynamic resuscitation was positive for methadone, THC, and benzodiazepines, but negative for cocaine, opiates, and amphetamines. Unfortunately, due to technical failure, the final urine sample was not sent for confirmatory mass spectrometry testing.

## 3. Discussion

This young, previously healthy patient died within six hours after smoking “Mr. Nice Guy,” with abrupt development of an abdominal catastrophe. The causative association with the use of this drug is very likely, specifically in light of the repeated episodes of transient abdominal pain following smoking of “Mr. Nice Guy” and the circumstances leading to his death, including hypertension following drug consumption, with a subsequent bout of abdominal pain, probably reflecting severe intestinal ischemia. Although the toxic screen upon admission was slightly positive for amphetamine, the final test was negative, thus ruling out cocaine and amphetamine as possible causative agents of the lethal intestinal ischemia. The initial positive test may be a false-positive or may suggest use of other illicit drugs prior to admission. Due to lack of patient cooperation, no additional screens were performed during hospitalization, to further evaluate these possibilities.

It is important to note that the patient was not treated with methadone or benzodiazepines by hospital staff, but rather with tramadol hydrochloride which acts as a weak opiate (though opiates were under the detection limit in the toxic screen done). This result is consistent with reports of the patient using illicit contraband drugs provided by visitors.

Conceivably, the repeated episodes of crampy abdominal pain throughout the hospitalization were caused by transient splanchnic arterial vasospasm, eventually leading to bowel necrosis and death. Indeed, in addition to their psychotropic effect, mediated mainly by CB1 and/or CB2 receptors in the brain, SCs have been shown to interfere with serotonergic and sympathomimetic neurotransmission, including induction of vasospasm, leading to a wide range of serious adverse reactions, such as kidney injury, pulmonary, gastrointestinal, neurological, and cardiovascular events [8, 18]. In a comprehensive review of more than 200 cases of SCs intoxication, serious medical complications developed within 24 hours of intake, including myocardial infarction, ischemic strokes, seizures, and acute kidney injury (AKI) [19]. Authors also report a wide range of psychoactive symptoms including agitation or irritability, restlessness, anxiety, confusion, short-term memory and cognitive impairment, and even psychosis. Other symptoms and physical findings reported include nausea and vomiting, slurred speech, chest pain and shortness of breath, dilated pupils, reddened conjunctivae, hypertension, tachycardia (up to 180 bpm), muscle twitches, excess sweating, and skin pallor. In this series laboratory tests and electrocardiogram (ECG) were generally unremarkable, except for mild leukocytosis (as in our case), hypokalemia, or hyperglycemia in some patients. Urine toxicological screens were often negative for illicit drugs.

Arterial vasospasm has been documented in some cases. We made a literature search (https://www.ncbi.nlm.nih.gov/pubmed/) using the phrases “synthetic cannabinoids” together with each of the following: “ischemia,” “vasospasm,” “thrombosis,” “perforation,” “intestinal,” and “abdomen.” Rose et al. reported two cases of subarachnoid hemorrhage following SCs consumption and used digital subtraction angiography to confirm transient vasospasm, suggesting a reversible cerebral vasoconstriction syndrome-like mechanism [8]. In an analysis of ninety-eight patients with cannabinoids-related strokes, reversible cerebral vasoconstriction triggered by cannabinoid use was shown to be a convincing mechanism of stroke in 27% of cases [20]. Mir et al. reported two patients with ST-elevation MI with normal coronary angiography [21] and Clark et al. reported eight pediatric patients with ECG changes compatible with myocardial ischemia [22]. Furthermore, gastrointestinal involvement was found as well. Buyukbese Sarsu described a young patient who developed duodenal perforation as a complication of a peptic ulcer disease, following chronic SC use [23]. Sevinc et al. reported a very rare case of acute gastric dilatation (AGD) and hepatic portal venous gas (HPVG) with findings of acute abdomen resulting from chronic use of a SC [24].

Conclusively, most of these reports illustrate that arterial vasospasm may be involved in some of the most serious complications of SCs abuse. However, to our knowledge, mesenteric events have not been reported so far. Yet, the medical course of our patient strongly suggests this possibility. In light of the above studies, vasospasm leading to intestinal ischemia and necrosis is a likely mechanism. SCs seem to be the causative agent, though we cannot exclude a role of the supplemental ingredients and dissolving materials present in his drug. We also cannot definitely exclude other etiologies for mesenteric ischemia, such as hypercoagulability, vasculitis, or arterial embolism, although the radiological findings suggest nonobstructive mesenteric ischemia. The patient's previous well-being, the lack of known familial coagulopathies, and the absence of other features of endocarditis or embolic phenomena (with the exception of a likely old splenic infarction, described in a former CT) make the hypothesis of mesenteric vascular spasm triggered by SCs consumption far more likely.

This case report suggests that fatal mesenteric ischemia may be added to a growing list of vascular complications of SCs. The fatal mesenteric event was likely preceded by transient episodes of mesenteric vasospasm, manifested by intense abdominal pain with leukocytosis and markers of inflammation, in the absence of other clinical abnormalities. We suggest that, in comparable settings, CT should better be performed with radio-contrast arterial enhancement. Further research is needed to elucidate the mechanisms of vasoconstriction associated with SCs intoxication.
